# Organizational environment as a factor of physicians’ psychological well-being

**DOI:** 10.1192/j.eurpsy.2021.1926

**Published:** 2021-08-13

**Authors:** M. Abdullaeva, G. Abdullayeva

**Affiliations:** 1 Psychology, Lomonosov Moscow State University, Moscow, Russian Federation; 2 Department Of Propaedeutic Of Childhood Diseases, Kazakh National Medical University after Asfendiyarov, Almaty, Kazakhstan

**Keywords:** well-being, physicians, “doctor - patient” relationship, public hospitals, commercial medical instit

## Abstract

**Introduction:**

The importance of ensuring the well-being of physicians is determined by the serious changes in medical organizations that transform the traditional “doctor - patient” relationship and set different indicators of the medical care quality (Melnyk et al., 2020; Sandy et al., 2019; Tawfik et al., 2019).

**Objectives:**

The main objective was to study the characteristics of the well-being of physicians working in public and commercial medical institutions. The difference in these “environments” is the degree of independence and responsibility in the course of diagnosis and treatment.

**Methods:**

The study involved 102 people: 66 of them are employees at public hospitals, 36 –at commercial medical centers. The respondents were offered a methodic package aimed to diagnose: career orientations; the degree of satisfaction with various work aspects; severity of burnout symptoms; subjective assessment of their work.

**Results:**

The estimating factor analysis identified 3 factors (73% of the total variance of the data) –such as emotional acceptance of one’s work, stress and tension, intellectual workload. The indicator of emotional exhaustion among physicians of commercial centers is significantly higher than that of doctors of public hospitals, which indicates a greater emotional involvement in the situation of providing paid services (p≤0.007).

**Conclusions:**

The main direction of psychological work with physicians of commercial institutions is teaching them to regulating the emotional state and to master communicative techniques. An important part of psychological support of physicians in public hospitals is to provide a favorable psychological climate that ensures the professional growth and adherence to humane principles of working with patients.

**Disclosure:**

No significant relationships.

